# Assessment and Impact of Cognitive Impairment in Multiple Sclerosis: An Overview

**DOI:** 10.3390/biomedicines7010022

**Published:** 2019-03-19

**Authors:** Miguel Ángel Macías Islas, Ethel Ciampi

**Affiliations:** 1 Department of Neurosciences, CUCS. University of Guadalajara and Mexico, Guadalajara 44160, Mexico; 2 Neurology, Pontificia Universidad Católica de Chile, Neurology, Hospital Dr. Sótero del Río, Santiago 8320000, Chile; ethelciampi@gmail.com

**Keywords:** multiple sclerosis, demyelinating diseases, cognitive impairment, cognitive dysfunction

## Abstract

Cognitive impairment affects 40–60% of patients with multiple sclerosis. It may be present early in the course of the disease and has an impact on a patient’s employability, social interactions, and quality of life. In the last three decades, an increasing interest in diagnosis and management of cognitive impairment has arisen. Neuropsychological assessment and neuroimaging studies focusing on cognitive impairment are now being incorporated as primary outcomes in clinical trials. However, there are still key uncertainties concerning the underlying mechanisms of damage, neural basis, sensitivity and validity of neuropsychological tests, and efficacy of pharmacological and non-pharmacological interventions. The present article aimed to present an overview of the assessment, neural correlates, and impact of cognitive impairment in multiple sclerosis.

## 1. Introduction

Multiple sclerosis (MS) is a chronic inflammatory demyelinating disease of the central nervous system (CNS). Typically, the disease affects the brain, spinal cord, and optic nerves, with acute inflammation as seen during MS relapses, and variable degrees of chronic inflammation and neurodegenerative processes within the white and gray matter, associated with progressive accumulation of disability. In about 85% of the patients, MS begins as a relapsing-remitting course and secondarily evolves to a progressive stage (secondary-progressive MS) in about 15–30% of patients [1,2]. From the onset, about 15% of the patients will develop a primary progressive course [3].

Most people experience their first symptoms of MS between the ages of 20 and 40. The clinical heterogeneity of MS, as well as the findings of different pathological patterns, suggests that MS may be a spectrum of diseases representing different processes [4,5,6,7]. MS can be clinically categorized in different phenotypes, including clinically isolated syndrome (CIS), relapsing/remitting (RRMS), primary progressive and secondary progressive MS (SPMS), and can be subclassified according to its clinical and radiological activity [8]. These phenotypes are related to potentially different pathophysiological disease mechanisms, including acute/chronic inflammation, axonal/neuronal loss and gliosis, and variable degrees of tissue repair, as well as plasticity and clinical recovery, mainly related to each individual [7], although these differences have yet to be demonstrated at the molecular level [2].

Clinical symptoms of MS may include motor dysfunction (pyramidal); tremor, dysmetria, or ataxia (cerebellar); diplopia or nystagmus (brainstem); numbness (sensory); urinary/bowel hesitancy, incontinence, or retention; disturbances in vision and cognitive impairment. The latter functional systems can be measured with the Expanded Disability Status Scale (EDSS), which range from 0 (normal neurological examination), to 10 (death due to MS) [9], and although it is the most widely used disability score worldwide, cognitive impairment related to the disease seems fairly underrepresented, even when cognitive and neuropsychiatric symptoms are a major cause of disability, loss of employment, and poor quality of life of patients and their families [10].

Although more than a century ago J.M. Charcot described “marked enfeeblement of the memory” with “conceptions that are formed slowly” in persons with “sclérose en plaques” [11], this elegant clinical observation was almost forgotten for more than a hundred years as a remarkable symptom of what is now known as MS. In 1991, S. Rao brought renewed attention to cognitive impairment in MS patients [12]. Since then, it has been a topic of clinical and basic research, trying to reveal the precise mechanisms behind its presentation, in order to develop effective treatments that include cognitive impairment as an outcome in clinical trials, many of them with unsatisfactory results [13].

The following manuscript is not a systematic review about the topic, but an overview that aims to raise awareness on the cognitive deficits in MS, including the most affected cognitive domains and related neuropsychological batteries for their assessment, their neural correlates with an emphasis on neuroimaging, and a potential therapeutic approach as well as future perspectives.

## 2. Cognitive Impairment in Multiple Sclerosis

Cognition represents the function of several neural pathways involved in the processing of information in the brain, including several correlated and interdependent cognitive domains such as executive function, perceptual-motor function, language, learning and memory, complex attention, and social cognition, as defined by the Diagnostic and Statistical Manual of Mental Disorders 5th edition (DSM-5) [14]. Impairment of individual domains may cause dysfunction of the global cognitive performance [15].

Although impairment in cognitive function occurs in different neurologic diseases, the clinical syndromes, the degree of dysfunction, and related disability, depend on the involvement of different brain structures (cortical or subcortical), the extent of neural damage or number of affected domains, and the patient’s previous cognitive reserve and performance. In MS, as a heterogeneous disease, all of the aforementioned characteristics makes it even more difficult to study cognition as a single manifestation of the disease [16,17].

Despite advances in knowledge about the neural basis of cognitive function in MS, there are still key uncertainties concerning what it is called ‘normal cognition’, and consequently with the assessment of cognitive dysfunction, typically defined as a performance below a chosen threshold in a number of cognitive domains, assessed in a specific neuropsychological test (e.g., 1.5–2 standard deviations below normal of a *Z*-score of one or more cognitive domains). In these batteries, results are commonly expressed as “intact/preserved” or “impaired” [18], and prevalent studies usually differ in cognitive impairment definitions.

Almost thirty years ago, in a population-based study performed by S. Rao et al., a 45% frequency of cognitive impairment in MS was found [12]. Other epidemiological studies reported frequencies of cognitive impairment in patients with MS between 40–70% in North America and Europe [19]. Frequencies of 40–60% have been reported in Latin America [20]. Even though a variety of different methodological approaches and neuropsychological batteries have been used, results are very similar across all reported populations.

MS is commonly diagnosed during a patient’s most productive life period, and employment years and cognitive impairment supposes a severe impact over a patient’s behavior, social functioning, adaptative strategies, and profound functional limitations affecting the activities of daily living and employment [10,21]. A large cross-sectional study carried out in nine European countries showed that only 35.8% of MS patients were employed. Low mood and cognitive impairment affecting domains like memory, attention, and slowed information processing speed were reported as frequent determinants of work-related difficulties, but only working memory impairment was responsible for higher unemployment rates [22]. Employment provides higher quality of life, independence, social participation, personal and professional reaffirmation, monetary income, health insurance, financial support for medication, and in some countries access to work benefits and social security [23], so cognition should be a priority in an era with highly active treatments reducing relapses and new lesions, and even new horizons in preventing accumulation of physical disability with new disease modifying treatments available [2].

A review by Shiavolin et al. concluded that difficulties that people with MS can experience with employment are always secondary outcomes of research, and it is quite difficult to address which factors contribute to reduced work participation. In their review, fatigue, mobility reduction, and cognitive impairment were reported as the main drivers of unemployment, and unemployment was related with worse quality of life scores [21].

In the same line, social cognition has gained relevance as a non-traditional cognitive domain present in MS since early stages of the disease, a domain that has been related to the capability for developing deep social interactions [24]. Recent evidence has shown 20% of social cognition impairment among patients, with a similar distribution for different phenotypes [25], and social cognition deficits show a significant correlation with the performance in other cognitive domains as working memory, processing speed, and executive functions [26,27,28] and exhibit behavioral impact affecting moral evaluation of other individuals’ actions [29].

Finally, cognitive impairment not only affects patients, but also affects their relationship with their families and is a frequent complaint of higher burden for caregivers [21]. Mickens et al. studied the mediational effect on the relationships between MS impairments (neurological, cognitive, behavioral, emotional, and functional), unmet family needs (household information, financial, social, support, and health), and caregiver mental health (satisfaction with life, anxiety, burden, and depression) using a structural equation model. They suggested that intervention research on MS caregivers in Latin America may consider focusing on caregiver mental health problems by addressing unmet family needs and teaching caregivers’ ways to manage the impairments of the individual with MS [30].

## 3. Cognitive Domains

All cognitive domains may be affected in MS, but the most affected ones are episodic memory and information processing speed [17,31]. Working memory, executive function, verbal fluency, and attention have also been widely described [12,32,33], with a recent interest in social cognition impairment [24,34]. Although clinical phenotypes may differ in the prevalence or severity of cognitive impairment, main determinants are physical disability as measured by EDSS, and patients’ age [35]. Other individual characteristics such as gender, genetic factors, and cognitive reserve may also play a relevant role [36]. For a summary of the most frequent cognitive domains affected in MS see Table 1.

### 3.1. Learning Memory

Long-term memory refers to the ability to learn new information and to recall that information at a later time point [39]. It is the most consistently affected cognitive domain in MS patients. Impaired learning of new information seems to be the primary problem [36], but the encoding, storing, and retrieval from long-term storage processes of memory seems to be affected in MS patients, so there is still controversy about which of these components of memory is the most influential factor for explaining memory deficits [40]. Other factors, such as slow processing speed, susceptibility to interference, executive disfunction, and perceptual deficits can also determine poor learning abilities [41].

### 3.2. Complex Attention—Information Processing

Complex attention domain involves sustained attention, divided attention, selective attention, and processing speed [42]. MS patients usually present with deficits in information processing efficiency, which refers to the ability to maintain and manipulate information in the brain for a short time period (working memory–executive function) [43] and to the speed with which one can process that information (processing speed–complex attention) [44]. It represents a key cognitive deficit in MS patients and might contribute to the presence of impairment in other cognitive domains [45,46].

### 3.3. Executive Function

Executive function is a complex domain which involves goal-directed behavior to adapt individuals to changes and demands of the environment, including planning, decision-making, working memory, responding to feedback, inhibition, and flexibility [42], and is affected in around 20% of MS patients. Some studies claim the difficulty to differentiate executive impairment from information processing, due to most of the tests used to evaluate executive function imply integrity of information processing and are affected by emotional affections such as depression. Leavitt et al. [47] studied executive functions and speed tasks (trail making test and Wisconsin card sorting test) in MS patients versus healthy controls. They found that MS patients score worse than controls, but differences decreased when corrected for information processing. They concluded that slow information processing accounts for executive function deficits in MS patients. The difficulty in assessing a specific domain, such as executive function, may be extrapolated to all other domains, as cognitive abilities are assessed individually in optimal environments, but patients usually struggle with managing multiple goals simultaneously [18].

### 3.4. Language

The language domain includes tasks such as object naming, word finding, fluency, grammar and syntax, and receptive language [42]. In MS, language deficits have been less studied than episodic memories or information processing speed. While some articles show intact functionality [48], more recent studies report frequencies of language impairment between 20% and 58% in RRMS or SPMS, respectively [38]. The most affected tasks seem to be phonological and semantic fluency, although verbal fluency tests are directly influenced by executive functions, thus many of the deficits have been considered as due to a dysfunctional executive syndrome [39].

### 3.5. Social Cognition

Social cognition, including social perception, empathy and theory of the mind, focuses on how people process, store, and apply information about other people and social situations, guiding social interactions [24]. Therefore, it is the sum of these processes that allow subjects of the same species to interact and exchange social codes to obtain information about another’s behavior, and about the environment [49]. Its recent inclusion within the six main cognitive domains of the DSM-5, and its association with quality of life and employment, have raised awareness among MS researchers in the last years [34].

Social perception has been defined as the ability to perceive information about the mental state of other subjects based on behavioral signals [50]. Empathy refers to the generation of an emotional response in the observer to situations affecting other subjects (e.g., same or different emotion), and it is an essential component of human emotional experience and social interaction, because when an observed mental state is understood, and affective responses are generated, prosocial and cooperative behaviors can exist [51,52]. Theory of the mind is the ability to represent the psychological perspective of interacting subjects, requiring an internal theorization about their thoughts and beliefs, emotions, affective states, and feelings [53].

Recent studies have shown 20–40% of social cognition impairment in MS patients, with similar distribution across phenotypes, greater impact in theory of the mind tasks, as well as in the recognition of certain negative facial emotion expressions [25,34]. It also seems that social cognition interacts with other cognitive domains, although a distinct patter of association with an exclusive domain (e.g., executive functions) has not been demonstrated [34,37].

## 4. Neuropsychological Assessment

Cognitive function assessment in MS patients should become a part of everyday clinical practice and as a constant outcome in clinical trials. Ideally, every patient with a diagnosis of MS should undergo a complete neuropsychological assessment and routinely repeat a standardized and validated battery to detect clinically meaningful changes, as well as start a timely and effective treatment, similar to what the Magnetic Resonance Imaging in MS (MAGNIMS) group has proposed for the MRI protocols in the diagnosis and monitoring of the disease [54,55]. Nonetheless, this desire from the cognitive research community has many obstacles, including key knowledge gaps and methodological limitations related to the understanding and measurement of cognitive deficits, neuroimaging of neural bases and correlations of deficits, as well as the development of effective treatments [18].

Mini-Mental State Examination by Folstein in 1975, which was used for dementia, is not sensitive to MS cognitive disorders [56]. The three most frequently used neurocognitive batteries in MS are: (1) The Brief Repeatable Battery of Neuropsychological tests (BRB-N), also known as Rao’s battery [57], (2) the minimal assessment of cognitive function in MS (MACFIMS) introduced by Benedict et al. [32], and (3) the Brief International Cognitive Assessment for Multiple Sclerosis (BICAMS), a shorter version that was developed in 2012 by an expert team, and is recommended for small centers with one or few staff members who may not have neuropsychological training [58]. All these screening batteries allow to establish the presence or absence of cognitive dysfunction and the specific domains affected. All of them have similarities and differences but share the fact that they are sensitive, specific, and cover the most frequently affected cognitive domains, and are also reasonably brief.

It is important to note that BICAMS should not be used within one month of recovery from relapse or within one month of steroid therapy, and the recommended order of administration is first the Symbol Digit Modalities Test (SDMT), then the California Verbal Learning Test (CVLT-II T1-5), and then the Brief Visuospatial Memory Test Revised (BVMT-R T1-3). In most clinical situations, yearly or bi-annual BICAMS evaluations will be appropriate.

Emphasis in testing MS cognitive impairment must be focused on the assessment of the most frequently affected domains, learning/memory, and information processing speed. In this context, experts are encouraging the MS multidisciplinary team (e.g., neurologists, nurses, psychologists, speech therapists, etc.) to be trained to use short MS cognitive assessment batteries, such as the BICAMS [12]. The subtests that compose the structure of domain specific evaluation of these batteries are shown in Table 2.

For the purpose of this review, we will describe the components of the BICAMS battery, due to its extensive use and validation in many countries, as well as the PASAT as being included as the cognitive test in MSFC, as well as a brief summary of social cognition tasks.

### 4.1. Information Processing Speed: Symbol Digit Modalities Test (SDMT)

When SDMT was first published in 1982, there were precedents of similar formats since 1927 and was adopted by the United States Army to assess precisely the speed of substitution of symbols by digits. SDMT has been used in almost every MS cognitive assessment battery and found to be exceptionally reliable and sensitive to assess information processing speed. The test consists of single digits paired with abstract symbols, with rows of the nine symbols arranged pseudo-randomly. The patient must say (or write) the number that corresponds with each symbol. The SDMT can be completed within 5 min, including instructions, practice, and testing. The SDMT has a reported sensitivity of 82% and a specificity of 60% [59]. It is the most sensitive task in MS, with good to excellent reliability, well tolerated by patients, has uniformity across languages, with no floor or ceiling effects, and a preliminary clinically meaningful change of 3–4 points [59], so it is recommended for clinical practice and research [18].

### 4.2. Episodic Verbal Memory: California Learning Verbal Test (CVLT)

This comprises of a 16-item word list, with four items belonging to each of the four categories, arranged randomly. The list is read aloud five times in the same order to the patient, at a slightly slower rate than one item per second. Patients are required to recall as many items as possible, in any order, after each reading. The CVLT-II T1-5 [60] can be completed in 5–10 min. It is recommended for clinical use, and it has high sensitivity with good age and sex adjusted normative data [18].

### 4.3. Episodic Visuospatial Memory: Brief Visuospatial Memory Test Revised (BVMT-R)

The BVMT-R T1-3 requires the patient to inspect a 2 × 3 stimulus array of abstract geometric figures. There are three learning trials of 10 s. The array is removed, and the patient is required to draw the array from memory, with the correct shapes in the correct position. It is also recommended for clinical and research use and has high sensitivity, it is time efficient, and is well tolerated by patients. Its main disadvantage is for patients with severe motor impairment [18].

### 4.4. Working Memory: Paced Auditory Serial Addition Test (PASAT-3”)

The PASAT is a measure of cognitive function that specifically assesses auditory information processing speed and flexibility, as well as calculation ability [61]. Stimulus presentation rates were adapted for use with MS patients by Rao and colleagues in 1989, and the measure has been widely used in MS studies since then. Single digits are presented either every 3” (or every 2” for the optional PASAT-2”, which could be a more accurate assessment of information processing speed) and the patient must add each new digit to the one immediately prior to it. The test score is the number of correct sums given (out of 60 possible sums) in each trial. To minimize familiarity with stimulus items in clinical trials and other serial studies, two alternate forms have been developed; the order of these should be counterbalanced across testing sessions [62,63]. Although it has been widely used in clinical research and clinical trials, and it has been included within the MSFC, there are several disadvantages to this test including a limited reliability due to practice effects, susceptibility to ceiling effect, poor tolerability due to a patient’s math ability, and test-related anxiety. Therefore, it is not recommended for cognitive monitoring in clinical practice, nor for clinical trials designed with multiple administrations, but it is better used as a putative cognitive processing task to compare results across previous studies [18].

### 4.5. Social Cognition

The assessment of social cognition in MS include a myriad of tests used in other neurological disorders, for example the Face and Emotion Recognition (e.g., Ekman faces [64]) for social perception, Faux Pas, or Reading the Mind in the Eyes tests for theory of the mind tasks, or compound batteries previously used in other neurological disorders such as in frontotemporal dementia (e.g., Social Emotion Assessment [65,66]). For example, the mini-Social and Emotional Assessment test (mini-SEA) includes the Faux Pas and the Face Emotion Recognition. The Faux Pas is comprised by ten narrative vignettes or short stories in which a character inadvertently hurts or offends another, using Theory of the Mind tasks to infer another’s mental state, making attributions to their knowledge, beliefs, and emotions. Half of the vignette test is control stories and the other half includes a principal character who inadvertently hurts or offends another, the ‘victim of the Faux Pas’. The subject is expected to recognize the situations in which a Faux Pas is committed, why the leading character did it (cognitive theory of mind, he did not mean it), and how the victim of the Faux Pas must have felt (affective theory of mind, we expect him to recognize that the victim must have had a negative emotion). The Face Emotion Recognition consists of 35 pictures for face affect recognition of basic emotions among a list presented at the bottom of the screen including happiness, sadness, anger, surprise, fear, disgust, and neutral [66].

There is still the need for a consensus statement from expert groups to select those tests with best sensitivity, specificity, and reliability in MS.

## 5. Neural Basis of Cognitive Impairment in MS

**Figure 1:**
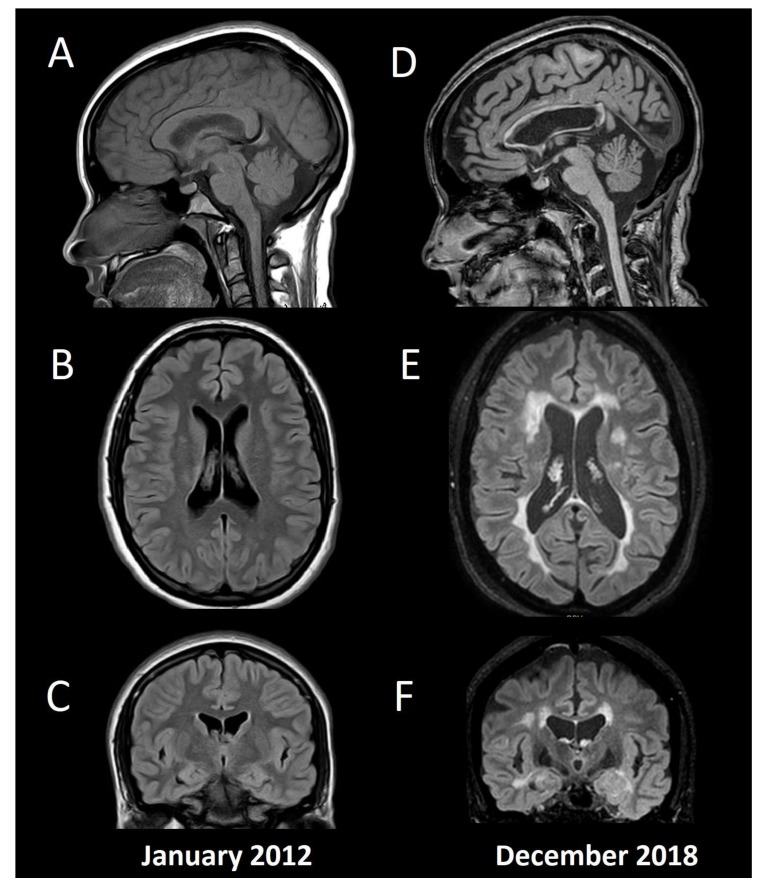
Conventional MRI in a patient with multiple sclerosis and cognitive impairment. Baseline MRI (**A**: Sagittal T1, **B**: Axial FLAIR, **C**: Coronal FLAIR) from a 15-year-old female with fulminant MS (Marburg variant, EDSS 8.0). After initial aggressive treatment in 2012, including myeloablation with cyclophosphamide, the patient remained asymptomatic without disease modifying treatment, until a second supratentorial motor relapse in 2015, confirming her MS diagnosis and beginning fingolimod. Since then, no relapses or new T2/enhancing lesions have appeared, and she had an EDSS 1.0 by the time of the second MRI in 2018 (**D**: Sagittal T1, **E**: Axial FLAIR, **F**: Coronal FLAIR). Her Mini-Mental State Examination was 30 (normal), Beck Depression Inventory 4 (without depression), and her fatigue severity score was 4 (significative fatigue). She had below normal performance (≤1.5 standard deviation) in verbal and visual episodic memory, and in information processing speed tests, with the diagnosis of cognitive impairment according to the MACFIMS battery. Note the widespread brain volume loss including cortical grey matter, ventricular width, and corpus callosum atrophy. The patient gave her written informed consent to present this data.

Underlying neural mechanisms of cognitive damage can be related to the inflammatory and neurodegenerative changes in the MS brain, including grey and white matter structures, both globally and regionally, structurally, and functionally [67]. Although one can appreciate some of these changes at a single-subject level (Figure 1), routine measurements (e.g., brain atrophy) are still not suggested to be used in clinical practice, mainly due to biological changes (e.g., dehydration, pseudo atrophy, etc.), that can exceed the accuracy threshold of current brain analysis software [55]. On the other hand, a myriad of group-analysis studies have been published trying to unveil the neural basis of cognitive impairment in MS. Differences in the results obtained by various studies may represent biased sample selection and differences between the image technology and software utilized in reported studies. Nonetheless, in vivo studies of neural correlations may contribute to early diagnosis, monitoring, and treatment of cognitive impairment in MS.

Structural imaging comprise of measurements of brain volume loss (atrophy), which can include global measurements, such as cross-sectional or longitudinal volumetric or 3D whole brain volume loss using semi-automated software (e.g., brain parenchymal fraction (BPF) [68], structural image evaluation using normalization of atrophy SIENAX—SIENA [69,70]), regional measurements of different tissues (e.g., grey matter or white matter volume, also measured by SIENAX), or specific grey or white matter structures (e.g., using voxel-based morphometry or Free Surfer for regional tissue volume loss or cortical thickness, respectively [71]), as well as manual/linear or 2D assessments such as the Corpus Callosum Index [72,73,74], or the third ventricle width, the frontal horn width, and the intercaudate distance [75].

From a functional point of view, although positron emission tomography (PET) and single photon emission computed tomography (SPECT) have shown correlations between cerebral blood flow and oxygen use with cognitive impairment in MS patients [76,77], functional MRI (fMRI) has gained its place among cognitive researchers in assessing the neural correlates of disability in MS, with an special emphasis on early changes, with a potential role for treatment monitoring (e.g., during cognitive rehabilitation) [78].

Also, recent interest has developed in the study of water diffusivity in normal-appearing white matter (regions of the white matter where no lesion is seen in conventional MRI studies), using diffusion tensor imaging (DTI), that can be related both with structural and functional disconnection between different regions of the brain [79]. Other non-conventional and advanced MRI techniques are also in study, including magnetization transfer imaging (MTI), proton MR spectroscopy (^1^H-MRS), and iron imaging [80].

Cognitive impairment has been associated with linear measure changes [75], the extent of white matter lesions and lesion load [29,33,37], focal cortical lesions [81], whole brain atrophy [82], diffuse cortical atrophy [81,83,84], regional grey matter structures such as thalamus, caudate, putamen, hippocampus, and amygdala, cerebellum and corpus callosum [67,85], as well as with widespread subtle pathological changes in normal-appearing white matter [83,86], among others.

Neuroanatomical correlates of memory deficits seem to differ across disease stages [87]. Early brain volume loss is a very precise predictor for the presence of cognitive impairment years later [82,83], and although hippocampal atrophy was not significantly seen in patients with CIS suggestive of MS compared with healthy controls in a study using DTI, hippocampal fractional anisotropy was significantly decreased in CIS patients, and mean diffusivity was correlated with verbal episodic memory performance [88]. An interesting study showed that a predictive model of cognitive performance in MS should include bilateral posterior cingulate cortex and bilateral temporal pole cortical thickness, overall white matter lesion load, normal-appearing white matter integrity, and age, reaffirming the multifactorial etiology of MS cognitive impairment [84].

Episodic memory has been correlated with total grey matter and regional cortical structures (e.g., left precuneus [89]); visuospatial memory has been associated with brain MRI total lesion area, T1 lesion, and FLAIR lesion volume, BPF, third ventricular width, and right superior frontal atrophy, among others [90,91]; verbal episodic memory has been associated with total and regional hippocampal atrophy, total lesion load and BPF [90,91,92,93]; information processing speed has been correlated with thalamus, whole grey matter atrophy, and third ventricle width [94], cerebellum atrophy [95,96], as well as with less white matter integrity, and increases in functional connectivity [79]; executive disfunction has been associated with frontal lobe structural and functional damage [97,98] and with dorsolateral prefrontal, orbitofrontal, anterior cingulate, and insular areas [99], as well as with thalamic structural and functional changes [100]; PASAT-3” scores have been correlated with cortical and subcortical structures such as bilateral precuneus, posterior cingulate, caudate putamen, and cerebellum [101], and acute changes in PASAT score with no physical changes (EDSS) have been associated with presence of acute gadolinium enhancing lesions [102], with similar results observe with transient SDMT changes [103], proposing that patients could also experience “cognitive relapses”.

Concerning social cognition, when assessing regional gray matter atrophy in a cohort of progressive MS patients with social cognitive impairment, significant loss was seen within bilateral cortical regions of orbitofrontal, insula and cerebellum, and right regions of fusiform gyrus, and precuneus [37], while functional MRI studies have shown increased activation in the posterior cingulate cortex and precuneus for the identification of anger and disgust faces, and greater activity in the occipital fusiform gyri, the anterior cingulate, and the inferior frontal cortex for the recognition of neutral faces [104]. Also, increased lesion volume has been correlated with lower success in face emotion recognition [105]. When assessing theory of the mind tasks, it seems that a disconnection syndrome, caused by white matter lesions, could also be one of the possible mechanisms underlying this specific impairment [105,106,107,108,109].

Other regions of interest associated with cognitive performance in multiple cognitive domains include the thalamus, as a relay station or cortico-subcortical and cortico-cortical networks [85] (e.g., global cognitive disfunction, information processing speed, attention, verbal memory, spatial memory, verbal fluency, executive function) [91,110,111,112]; the cerebellum, as a historically understudied region for cognitive performance (attention, working memory, information processing speed, etc.) [37,95,96]; and the corpus callosum, the main white matter tract of the brain (e.g., information processing speed, working memory, verbal fluency, etc.) [73,74].

Finally, we would like to highlight advances of fMRI and cognitive research in MS [78], although there has been some controversies about the real meaning of fMRI results, it seems that early changes can be seen, even in cognitively preserved patients, including higher recruitment of non-related areas, such as supplementary motor cortex during working memory tasks [113] or by changes in activity properties of regions highly related to cognitive functions, as centrality measures of the default mode network [114], changes that may be used as a biomarker for neurocognitive rehabilitation [115] especially, resting state fMRI [116,117,118].

## 6. Treatment of Cognitive Impairment in MS

### 6.1. Pharmacological Interventions

#### 6.1.1. MS Disease Modifying Therapies

MS specific disease modifying therapies such as the injectables interferon beta, glatiramer acetate; oral agents such as fingolimod, teriflunomide, or dimethyl fumarate; and monoclonal antibodies such as natalizumab, alemtuzumab, and ocrelizumab, have shown significant benefits in reducing the annualized relapse rate and MRI activity (new T2 or gadolinium enhancing lesions), with a more discrete efficacy over reducing disability progression or the brain atrophy rate [2]. However, their specific impact on cognitive impairment remains unclear, mainly because most phase III clinical trials established cognitive impairment as a secondary or tertiary outcome measure. Comparative efficacy on cognitive outcomes across trials is even more difficult, because of the different neuropsychological batteries used, the varied methods for evaluation and outcome analysis, and the differences between populations included in the trials.

Pivotal interferons and glatiramer acetate clinical trials did not include cognitive evaluation as primary endpoints. Intramuscular interferon beta 1a versus placebo included neuropsychological evaluation as a secondary outcome measure and showed 52.7% improvement compared with 29% in the placebo group [119], including processing speed and episodic memory outcomes. In the COGIMUS (Cognitive Impairment in Multiple Sclerosis) study, subcutaneous interferon 1a protected RRMS patients from general cognitive decline when reevaluated at 3 [120] and 5 years [121] after therapy onset. Regarding interferon beta 1b, Pishkin reported only improvement of delayed visual reproduction performance [122], and the Betaferon/Betaseron in Newly Emerging Multiple Sclerosis for Initial Treatment (BENEFIT) trial revealed that in patients with CIS interferon beta 1b had beneficial effects on working memory, and the effects remained over 8 years [123]. Glatiramer Acetate trials, while included BRB-N evaluation, did not show significant differences versus placebo [62].

The GOLDEN Study using once-daily oral fingolimod was compared with interferon beta 1b using a trial design that included cognitive impairment as the primary outcome. This study showed improvement in cognitive function (BRB-N and DKEFS) in both treatment arms, favoring fingolimod on MRI parameters [69], although some baseline imbalances may have favored the interferon beta 1b arm, according to the authors. In a patient-reported outcomes study, evaluating global satisfaction in switching treatment from several disease modifying drugs to teriflunomide and using SDMT to measure cognitive impairment, results showed that patients and physicians reported stability of cognition in a 48-week period [124].

Natalizumab pivotal studies showed that it can reduce the risk of progressive working memory impairment by 43% compared with placebo [125]. In a long-term observational study by Jacques et al., natalizumab was reported to preserve cognition over 7 years of continuous therapy using a computed test and the SDMT. No patient showed evidence of sustained cognitive deterioration over a 24-month period [126]. In a study including 21 patients during a 15-month follow-up period, alemtuzumab showed stable cognitive function using an extensive neuropsychological battery [127]**.** Ocrelizumab has shown improvement in MS Functional Composite score (a composite measure of walking speed, upper-limb movements, and cognition assessed by PASAT) compared with interferon beta 1a [128].

#### 6.1.2. Cognitive Impairment-Specific Treatment

The use of acetylcholinesterase inhibitors (AChEI) in multiple sclerosis patients remains controversial. Few studies in a small number of MS patient populations reported contradictory results. While Krupp in 2004 reported the positive impact of donepezil in verbal learning and memory in a cohort of 69 patients [129]**,** the same investigator reported no significant effect in 2011, which included 120 MS patients [130]. It is important to stress that long term treatments with AChEI compels one to be aware of the side effects. Regarding memantine, similar contradictory findings were reported in a small number of studies prevailing negative outcomes for this drug [131].

Amphetamines significantly improved visuospatial memory and verbal memory [132], fampridine has shown to be able to improve cognitive fatigue, alertness, psychomotor speed, and verbal fluency [133,134], while no benefit on learning were found using modafinil [135].

### 6.2. Non-Pharmacological Interventions

#### 6.2.1. Neuropsychological Rehabilitation

Only recently, neuropsychological rehabilitation has been established as a useful therapeutic tool. Multidisciplinary and cognitive-behavioral interventions, computer-assisted training and combinations of the above, have been showing consistently better results [13,136], especially when tailored to individual needs. Evidence-based conclusions have only recently become stronger in regards to which interventions may have real benefits for MS patients. In a recent review article and meta-analysis including literature from 2007 to 2016, only one intervention received support for a practice standard in verbal learning and memory (modified Story Memory Technique—mSMT [137]), two computer programs received support as a practice guideline for attention and multicognitive domains (Attention Process Training—APT [138] and RehaCom [139]), and several studies provided support for the practice option in attention, learning, and memory [140].

#### 6.2.2. Exercise

To date, numerous publications have shown the positive impact of physical exercise on different clinical parameters, but evidence remains to be demonstrated, as clinical trials have shown equivocal results [141,142]. A systematic review by Sandroff et al. showed that a few comparable studies did not yield a significant positive impact of physical exercise on cognitive impairment outcomes [143]. Another systematic review of the impact of yoga also failed to show the effect of this discipline in cognitive impairment [144]. This maybe the result of collectively insufficient well–designed research, and again, the fact that cognitive impairment is maintained as a secondary outcome. The cognitive effects of physical exercise in MS is gaining hype among cognitive researchers, as one relevant intervention both in preventing and improving cognitive outcomes, although clear results, as well as doses and regimens (e.g., aerobic versus weight training), are still missing [13].

## 7. Conclusions

In the last three decades, increasing knowledge in the field of cognitive impairment in MS has arisen. From defining the most sensitive neuropsychological tests and compound batteries for clinical practice and research, to better understanding the neural correlates in specific populations with assistance from conventional-structural and non-conventional/functional neuroimaging, better and more effective treatment, rehabilitation, and prevention strategies are being proposed.

Cognitive function assessment should be included in the standard clinical evaluation and clinical trials involving MS patients, and treatment strategies should be implemented as supported by current evidence. Limitations are still present, especially due to the validation and standardization of both diagnostic and therapeutic tools.

Due to the devastating impact over the working status, social interaction, and self-care of MS patients, improvement in all the aforementioned areas, as well as education to patients, families, and the community should be stated as a priority, and an unmet need.

## Figures and Tables

**Table 1:** Frequency of cognitive impairment in patients with multiple sclerosis (MS) by cognitive domain.

Cognitive Domain	Frequency
**Learning Memory**	**40–65%**
*Visual Episodic Memory*	20–75%
*Verbal Episodic Memory*	15–80%
**Complex Attention**	**5–25%**
*Information processing Speed*	15–50%
**Executive Function**	**15–25%**
*Working Memory*	15–60%
*Inhibitory control*	15–30%
**Language**	**20–58%**
*Verbal Fluency*	15–25%
**Social Cognition**	**20–40%**

MS: Multiple sclerosis. Adapted from Rao et al. 1991 [12], Benedict et al. 2006 [32], Chiaravaloti et al. 2008 [17], Dulau 2017 [25], Cotter et al. 2018 [34], Ciampi et al. 2018 [37], Ntoskou et al. 2018 [38].

**Table 2:** Comparison of the three most used neuropsychological batteries in MS.

Cognitive Domain	BRB-N	MACFIMS	BICAMS
Auditory processing speed and working memory	PASAT	PASAT	-
Visual processing speed and working memory	SDMT	SDMT	SDMT
Auditory or verbal episodic memory	SRT	CVLT-II	CVLT-II
Visual or spatial episodic memory	10/36 Spatial Recall Test	BVMT-R	BVMT-R
Expressive language	COWAT	COWAT	-
Spatial processing	-	JLO	-
Executive function	-	DKEFS sorting	-

MS: Multiple sclerosis, BRB-N: Brief Repeatable Battery of Neuropsychological tests, MACFIMS: Minimal assessment of cognitive function in MS, BICAMS: Brief International Cognitive Assessment for Multiple Sclerosis, PASAT: Paced Auditory Serial Addition, SDMT: Symbol Digit Modalities Test, SRT: Selective Reminding Test, CVLT-II: California Verbal Learning Test, BVMT-R: Brief Visuospatial Memory Test Revised, COWAT: Controlled Oral Word Association Test, JLO: Judgement of Line Orientation test, DKEFS: Delis-Kaplan Executive Function System.

## References

[1] Cree B.A., Gourraud P.A., Oksenberg J.R., Bevan C., Crabtree-Hartman E., Gelfand J.M., Goodin D.S., Graves J., Green A.J., Mowry E. (2016). Long-term evolution of multiple sclerosis disability in the treatment era. Ann. Neurol..

[2] Thompson A.J., Baranzini S.E., Geurts J., Hemmer B., Ciccarelli O. (2018). Multiple sclerosis. Lancet.

[3] Miller D.H., Leary S.M. (2007). Primary-progressive multiple sclerosis. Lancet Neurol..

[4] Kakalacheva K., L€unemann J.D. (2011). Enviromental triggers of multiple sclerosis. FEBS Lett..

[5] Chitnis T. (2013). Role of puberty in multiple sclerosis risk and course. Clin. Immunol..

[6] Franciotta D., Salvetti M., Lolli F., Serafini B., Aloisi F. (2008). B cells and multiple sclerosis. Lancet Neurol..

[7] Fletcher J.M., Lalor S.J., Sweeney C.M., Tubridy N., Mills K.H.G., Lalor S. (2010). T cells in multiple sclerosis and experimental autoimmune encephalomyelitis. Clin. Exp. Immunol..

[8] Lublin F.D., Reingold S.C., Cohen J.A., Cutter G.R., Sørensen P.S., Thompson A.J., Wolinsky J.S., Balcer L.J., Banwell B., Barkhof F. (2014). Defining the clinical course of multiple sclerosis: The 2013 revisions. Neurology.

[9] Kurtzke J.F. (1983). Rating neurologic impairment in multiple sclerosis: An expanded disability status scale (EDSS). Neurology.

[10] Clemens L., Langdon D. (2018). How does cognition relate to employment in multiple sclerosis? A systematic review. Mult. Scler. Relat. Disord..

[11] Murray J. (2005). Terminology and disease description. Multiple Sclerosis the History of a Disease.

[12] Rao S., Leo G., Bernardin I., Unverzagt F. (1991). Cognitive dysfunction in multiple sclerosis: Frequency, patterns, and predictions. Neurology.

[13] Sokolov A.A., Grivaz P., Bove R. (2018). Cognitive Deficits in Multiple Sclerosis: Recent Advances in Treatment and Neurorehabilitation. Curr. Treat. Options Neurol..

[14] American Psychiatric Association (2013). Diagnostic and Statistical Manual of Mental Disorders.

[15] Wooddruff B.K. (2011). Disorders of cognition. Semin. Neurol..

[16] Amato P., Langdon D., Montalvan X., Benedict R., DeLuca J., Krupp L.B., Thompson A.J., Comi G. (2013). Treatment of cognitive impairment in multiple sclerosis: Position paper. J. Neurol..

[17] Chiaravaloti N.D., DeLuca J. (2008). Cognitive impairment in multiple sclerosis. Lancet.

[18] Sumowski J.F., Benedict R., Enzinger C., Filippi M., Geurts J.J., Hamalainen P., Hulst H., Inglese M., Leavitt V.M., Rocca M.A. (2018). Cognition in multiple sclerosis, State of the field and priorities for the future. Neurology.

[19] DiGiuseppe G., Blair M., Morrow S.A. (2018). Prevalence of cognitive impairment in newly diagnosed relapsing-remitting multiple sclerosis. Int. J. MS Care.

[20] Vanotti S., Caceres F.J. (2017). Cognitive and neuropsychiatric disorders among MS patients from Latin America. MSJ Exp. Trans. Clin..

[21] Schiavolin S., Leonardi M., Giovannetti A.M., Antozzi C., Brambilla L., Confalonieri P., Mantegazza R., Raggi A. (2013). Factors related to difficulties with employment in patients with multiple sclerosis: A review of 2002–2011 literature. Int. J. Rehabil. Res..

[22] Raggi A., Covelli V., Schiavolin S., Scaratti C., Leonardi M., Willems M. (2016). Work-related problems in multiple sclerosis: A literature review on its associates and determinants. Disabil. Rehabil..

[23] Yamout B., Issa Z., Herlopian A., El Bejjani M., Khalifa A., Ghadieh A.S., Habib R.H. (2013). Predictors of quality of life among multiple sclerosis patients: A comprehensive analysis. Eur. J. Neurol..

[24] Labbé T., Ciampi E., Carcamo Rodríguez C. (2018). Social cognition: Concepts, neural basis and its role in multiple sclerosis. Neurol. Clin. Neurosci..

[25] Dulau C., Deloire M., Diaz H., El Bejjani M., Khalifa A., Ghadieh A.S., Habib R.H. (2017). Social cognition according to cognitive impairment in different clinical phenotypes of multiple sclerosis. J. Neurol..

[26] Raimo S., Trojano L., Pappacena S., Alaia R., Spitaleri D., Grossi D., Santangelo G. (2017). Neuropsychological Correlates of Theory of Mind Deficits in Patients with Multiple Sclerosis. Neuropsychology.

[27] Carotenuto A., Arcara G., Orefice G., Cerillo I., Giannino V., Rasulo M., Iodice R., Bambini V. (2017). Communication in Multiple Sclerosis: Pragmatic Deficit and its Relation with Cognition and Social Cognition. Arch. Clin. Neuropsychol..

[28] Henry A., Tourbah A., Chaunu M.P., Bakchine S., Montreuil M. (2017). Social Cognition Abilities in Patients with Different Multiple Sclerosis Subtypes. J. Int. Neuropsychol. Soc..

[29] Patil I., Young L., Sinay V., Gleichgerrcht E. (2017). Elevated moral condemnation of third-PARTY violations in multiple sclerosis patients. Soc. Neurosci..

[30] Mickens M.N., Perrin P.B., Aguayo A., Rabago B., Macias-Islas M., Arango-Lasprilla J. (2018). Mediational Model of Multiple Sclerosis Impairments, Family Needs, and Caregiver Mental Health in Guadalajara, Mexico. Behav. Neurol..

[31] Langdon D.W. (2011). Cognition in multiple sclerosis. Curr. Opin. Neurol..

[32] Benedict R.H., Cookfair D., Gavett R., Gunther M., Munschauer F., Garg N., Weinstock-Guttman B. (2006). Validity of the Minimal Assessment of Cognitive Function in Multiple Sclerosis (MACFIMS). J. Int. Neuropsychol. Soc..

[33] Deloire M.S., Ruet A., Hamel D., Bonnet M., Dousset V., Brochet B. (2011). MRI predictors of cognitive outcome in early multiple sclerosis. Neurology.

[34] Cotter J., Firth J., Enzinger C., Kontopantelis E., Yung A.R., Elliott R., Drake R.J. (2016). Social cognition in multiple sclerosis: A systematic review and meta-analysis. Neurology.

[35] Ruano L., Portaccio E., Goretti B., Niccolai C., Severo M., Patti F., Cilia S., Gallo P., Grossi P., Ghezzi A. (2017). Age and disability drive cognitive impairment in multiple sclerosis across disease subtypes. Mult. Scler..

[36] Trenova A.G., Slavov G.S., Manova M.G., Aksentieva J.B., Miteva L.D., Stanilova S.A. (2016). Cognitive impairment in multiple sclerosis. Folia Med..

[37] Ciampi E., Uribe-San-Martin R., Vásquez M., Ruiz-Tagle A., Labbe T., Cruz J.P., Lillo P., Slachevsky A., Reyes D., Reyes A. (2018). Relationship between Social Cognition and traditional cognitive impairment in Progressive Multiple Sclerosis and possible implicated neuroanatomical regions. Mult. Scler. Relat. Disord..

[38] Ntoskou K., Messinis L., Nasios G., Martzoukou M., Makris G., Panagiotopoulos E., Papathanasopoulos P. (2018). Cognitive and Language Deficits in Multiple Sclerosis: Comparison of Relapsing Remitting and Secondary Progressive Subtypes. Open Neurol. J..

[39] Lezak M.D., Howieson D.B., Bigler E.D., Tranel D. (2012). Neuropsychological Assessment.

[40] Thornton A.E., Raz N., Tucke K.A. (2002). Memory in multiple sclerosis: Contextual encoding defi cits. J. Int. Neuropsychol. Soc..

[41] Renell P.G., Jensen F., Henry J.D. (2007). Prospective memory in multiple sclerosis. J. Int. Neuropsychol. Soc..

[42] Sachdev P.S., Blacker D., Blazer D.G., Ganguli M., Jeste D.V., Paulsen J.S., Petersen R.C. (2014). Classifying neurocognitive disorders: The DSM-5 approach. Nat. Rev. Neurol..

[43] Silva P.H.R., Spedo C.T., Baldassarini C.R., Benini C.D., Ferreira D.A., Barreira A.A., Leoni R.F. (2019). Brain functional and effective connectivity underlying the information processing speed assessed by the Symbol Digit Modalities Test. Neuroimage.

[44] Costa S.L., Genova H.M., DeLuca J., Chiaravalloti N.D. (2016). Information processing speed in multiple sclerosis: Past, present, and future. Mult. Scler. J..

[45] Grzegorski T., Losy J. (2017). Cognitive impairment in multiple sclerosis. Rev. Neurosci..

[46] Van Schependom J., D’hooghe M.B., Cleynhens K., D’hooge M., Haelewyck M.C., De Keyser J., Nagels G. (2015). Reduced information processing speed as primum movens for cognitive decline in MS. Mult. Scler..

[47] Leavitt V.M., Wylie G., Krch D., Chiaravalloti N., DeLuca J., Sumowski J.F. (2014). Does slowed processing speed account for executive deficits in multiple sclerosis Evidence from neuropsychological performance and structural neuroimaging. Rehabil. Psychol..

[48] Prakash R.S., Snook E.M., Lewis J.M., Motl R.W., Kramer A.F. (2008). Cognitive impairments in relapsing-remitting multiple sclerosis: A meta-analysis. Mult. Scler..

[49] Frith C.D., Frith U. (2007). Social cognition in humans. Curr. Biol..

[50] De Bruin L., Strijbos D. (2015). Direct social perception, mindreading and Bayesian predictive coding. Conscious Cogn..

[51] Singer T., Lamm C. (2009). The social neuroscience of empathy. Ann. N. Y. Acad. Sci..

[52] Ruggieri V.L. (2013). Empathy, social cognition and autism spectrum disorders. Rev. Neurol..

[53] Baron-Cohen S. (1988). Without a theory of mind one cannot participate in a conversation. Cognition.

[54] Rovira À., Wattjes M.P., Tintoré M., Tur C., Yousry T.A., Sormani M.P., De Stefano N., Filippi M., Auger C., Rocca M.A. (2015). Evidence-based guidelines: MAGNIMS consensus guidelines on the use of MRI in multiple sclerosis-clinical implementation in the diagnostic process. Nat. Rev. Neurol..

[55] Wattjes M.P., Rovira À., Miller D., Yousry T.A., Sormani M.P., de Stefano M.P., Tintoré M., Auger C., Tur C., Filippi M. (2015). Evidence-based guidelines: MAGNIMS consensus guidelines on the use of MRI in multiple sclerosis—Establishing disease prognosis and monitoring patients. Nat. Rev. Neurol..

[56] Beatty W.W., Goodkin D.E. (1990). Screening for cognitive impairment in multiple sclerosis. An evaluation of the Mini-Mental State Examination. Arch. Neurol..

[57] Rao S.M. (1991). Neuropsychological Screening Battery for Multiple Sclerosis.

[58] Langdon D.W., Amato M.P., Boringa J., Brochet B., Foley F., Fredrikson S., Hämäläinen P., Hartung H.-P., Krupp L., Penner I.K. (2012). Recommendations for a Brief International Cognitive Assessment for Multiple Sclerosis (BICAMS). MSJ.

[59] Benedict R., DeLuca J., Phillips G., LaRocca N., Hudson L.D., Rudick R., Multiple Sclerosis Outcome Assessments Consortium (2017). Validity of the Symbol Digit Modalities Test as a cognition performance outcome measure formultiple sclerosis. Mult. Scler. J..

[60] Delis D.C., Kramer J.H., Kaplan E., Ober B.A. (2000). California Verbal Learning Test.

[61] Gronwall D. (1977). Paced auditory serial addition task: A measure of recovery from concussion. Percept. Motor Skills.

[62] Cutter G.R., Baier M.S., Rudick R.A., Cookfair D.L., Fischer J.S., Petkau J., Syndulko K., Weinshenker B.G., Antel J.P., Confavreux C. (1999). Development of a Multiple Sclerosis Functional Composite as a clinical trial outcome measure. Brain.

[63] Fischer J.S., Rudick R.A., Cutter G.R., Reingold S.C. (1999). The Multiple Sclerosis Functional Composite Measure (MSFC): An integrated approach to MS clinical outcome assessment. National MS Society Clinical Outcomes Assessment Task Force. Mult. Scler..

[64] Friesen W.V., Ekman P. (1976). Friesen Pictures of Facial Affect.

[65] Funkiewiez A., Bertoux M., de Souza L.C., Lévy R., Dubois B. (2012). The sea (social cognition and emotional assessment): A clinical neuropsychological tool for early diagnosis of frontal variant of frontotemporal lobar degeneration. Neuropsychology.

[66] Bertoux M., Volle E., de Souza L.C., Funkiewiez A., Dubois B., Habert M.O. (2014). Neural correlates of the mini-SEA (Social cognition and Emotional Assessment) in behavioral variant frontotemporal dementia. Brain Imaging Behav..

[67] Di Filippo M., Portaccio E., Mancini A., Calabresi P. (2018). Multiple sclerosis and cognition: Synaptic failure and network dysfunction. Nat. Rev. Neurosci..

[68] Zivadinov R., Sepcic J., Nasuelli D., De Masi R., Bragadin L.M., Tommasi M., Zambito-Marsala S., Moretti R., Bratina A., Ukmar M. (2001). A longitudinal study of brain atrophy and cognitive disturbances in the early phase of relapsing-remitting multiple sclerosis. J. Neurol. Neurosurg. Psychiatry.

[69] Smith S.M., Zhang Y., Jenkinson M., Chen J., Matthews P.M., Federico A., de Stefano N. (2002). Accurate, robust and automated longitudinal and cross-sectional brain change analysis. NeuroImage.

[70] Smith S.M., Jenkinson M., Woolrich M.W., Beckmann C.F., Behrens T.E.J., Johansen-Berg H., Bannister P.R., de Luca M., Drobnjak I., Flitney D.E. (2004). Advances in functional and structural MR image analysis and implementation as FSL. NeuroImage.

[71] Geurts J.G., Calabrese M., Fisher E., Rudick R.A. (2012). Measurement and clinical effect of grey matter pathology in multiple sclerosis. Lancet Neurol..

[72] Figueira F.F., Santos V.S., Figueira G.M.A., Da Silva Â.C.M. (2007). Corpus callosum index: A practical method for long-term follow-up in multiple sclerosis. Arq. Neuropsiquiatr..

[73] Yaldizli Ö., Penner I.K., Frontzek K., Naegelin Y., Amann M., Papadopoulou A., Sprenger T., Kuhle J., Calabrese P., Radü E.W. (2014). The relationship between total and regional corpus callosum atrophy, cognitive impairment and fatigue in multiple sclerosis patients. Mult. Scler..

[74] Uribe-San-Martín R., Ciampi E., Di Giacomo R., Vásquez M., Cárcamo C., Godoy J., Lo Russo G., Tassi L. (2018). Corpus callosum atrophy and post-surgical seizures in temporal lobe epilepsy associated with hippocampal sclerosis. Epilepsy Res..

[75] Butzkueven H., Kolbe S.C., Jolley D.J., Brown J.Y., Cook M.J., van der Mei I.A., Groom P.S., Carey J., Eckholdt J., Rubio J.P. (2008). Validation of linear cerebral atrophy markers in multiple sclerosis. J. Clin. Neurosci..

[76] Lycke J., Wikkelso C., Bergh A.C., Jacobsson L., Andersen O. (1993). Regional cerebral blood flow in multiple sclerosis masured by single photon emission tomography with technetium-99m hexamethylpropyleneamine oxime. Eur. Neurol..

[77] Roelcke U., Kappos L., Lechner-Scott J., Brunnschweiler H., Huber S., Ammann W., Plohmann A., Dellas S., Maguire R.P., Missimer J. (1997). Reduced glucose metabolism in the frontal cortex and basal ganglia of multiple sclerosis patients with fatigue a 18F-fl uorodeoxyglucose positron emission tomography study. Neurology.

[78] Labbé T., Ciampi E., Cruz J.P., Zurita M., Uribe S., Cárcamo C. (2018). Functional magnetic resonance imaging in the study of multiple sclerosis. Rev. Neurol..

[79] Meijer K.A., Muhlert N., Cercignani M., Sethi V., Ron M.A., Thompson A.J., Miller D.H., Chard D., Geurts J.J., Ciccarelli O. (2016). White matter tract abnormalities are associated with cognitive dysfunction in secondary progressive multiple sclerosis. Mult. Scler..

[80] Giorgio A., De Stefano N. (2016). Advanced Structural and Functional Brain MRI in Multiple Sclerosis. Semin. Neurol..

[81] Calabrese M., Agosta F., Rinaldi F., Mattisi I., Grossi P., Favaretto A., Atzori M., Bernardi V., Barachino L., Rinaldi L. (2009). Cortical lesions and atrophy associated with cognitive impairment in relapsing-remitting multiple sclerosis. Arch. Neurol..

[82] Summers M.M., Fisniku L.K., Anderson V.M., Miller D.H., Cipolotti I., Ron M. (2008). Cognitive impairment in relapsing remitting multiple sclerosis can be predicted by imaging performed several years earlier. Mult. Scler..

[83] Tekok-Kilic A., Benedict R.H., Weinstock-Gutman B., Dwyer M.G., Carone D., Srinivasaraghavan B., Yella V., Abdelrahman N., Munschauer F., Bakshi R. (2007). Independent contributions of cortical grey matter atrophy and ventricle enlargement for predicting neuropsychological impairment in multiple sclerosis. Neurology.

[84] Steenwijk M.D., Geurts J.J.G., Daams M., Tijms B.M., Wink A.M., Balk L.J., Tewarie P.K., Uitdehaag B.M.J., Barkhof F., Vrenken H. (2016). Cortical atrophy patterns in multiple sclerosis are non-random and clinically relevant. Brain.

[85] Kern K.C., Gold S.M., Lee B., Montag M., Horsfall J., O’Connor M.F., Sicotte N.L. (2014). Thalamic-hippocampal-prefrontal disruption in relapsing-remitting multiple sclerosis. Neuroimage Clin..

[86] Zivadinov R., de M.R., Nasuelli D., Bragadin L.M., Ukmar M., Pozzi-Mucelli R.S., Grop A., Cazzato G., Zorzon M. (2001). MRI techniques and cognitive impairment in the early phase of relapsing-remitting multiple sclerosis. Neuroradiology.

[87] Sumowski J.F., Leavitt V.M., Rocca M.A., Inglese M., Riccitelli G., Buyukturkoglu K., Meani A., Filippi M. (2018). Mesial temporal lobe and subcortical grey matter volumes differentially predict memory across stages of multiple sclerosis. Mult. Scler..

[88] Planche V., Ruet A., Coupé P., Lamargue-Hamel D., Deloire M., Pereira B., Manjon J.V., Munsch F., Moscufo N., Meier D.S. (2017). Hippocampal microstructural damage correlates with memory impairment in clinically isolated syndrome suggestive of multiple sclerosis. Mult. Scler..

[89] Aladro Y., López-Alvarez L., Sánchez-Reyes J.M., Hernández-Tamames J.A., Melero H., Rubio-Fernández S., Thuissard I., Cerezo-García M. (2018). Relationship between episodic memory and volume of the brain regions of two functional cortical memory systems in multiple sclerosis. J. Neurol..

[90] Benedict R.H., Bakshi R., Simon J.H., Priore R., Miller C., Munschauer F. (2002). Frontal cortex atrophy predicts cognitive impairment in multiple sclerosis. J. Neuropsych. Clin. Neurosci..

[91] Houtchens M.K., Benedict R.H., Kiliiany R., Sharma J., Jaisani Z., Singh B., Weinstock-Guttman B., Guttmann C.R., Bakshi R. (2007). Thalamic atrophy and cognition in multiple sclerosis. Neurology.

[92] Sicotte N.L., Kern K.C., Giesser B.S., Arshanapalli A., Schultz A., Montag M., Wang H., Bookheimer S.Y. (2008). Regional hippocampal atrophy in multiple sclerosis. Brain.

[93] González Torre J.A., Cruz Gómez Á.J., Belenguer A., Sanchis-Segura C., Ávila C., Forn C. (2017). Hippocampal dysfunction is associated with memory impairment in multiple sclerosis: A volumetric and functional connectivity study. Mult. Scler..

[94] Minagar A., Barnett M.H., Benedict R.H.B., Pelletier D., Pirko I., Sahraian M.A., Frohman E., Zivadinov R. (2013). The thalamus and multiple sclerosis: Modern views on pathologic, imaging, and clinical aspects. Neurology.

[95] Ruet A., Hamel D., Deloire M.S., Charré-Morin J., Saubusse A., Brochet B. (2014). Information processing speed impairment and cerebellar dysfunction in relapsing-remitting multiple sclerosis. J. Neurol. Sci..

[96] Moroso A., Ruet A., Lamargue-Hamel D., Munsch F., Deloire M., Coup P., Ouallet J.C., Planche V., Moscufo N., Meier D.S. (2017). Posterior lobules of the cerebellum and information processing speed at various stages of multiple sclerosis. J. Neurol. Neurosurg. Psychiatry.

[97] Foong J., Rozewicz L., Quaghebeur G., Davie C.A., Kartsounis L.D., Thompson A.J., Miller D.H., Ron M.A. (1997). Executive function in multiple sclerosis. The role of frontal lobe pathology. Brain.

[98] Muhlert N., Sethi V., Schneider T., Daga P., Cipolotti L., Haroon H.A., Parker G.J.M., Ourselin S., Wheeler-Kingshott C.A.M., Miller D.H. (2013). Diffusion MRI-based cortical complexity alterations associated with executive function in multiple sclerosis. J. Magn. Reson. Imaging.

[99] Weygandt M., Wakonig K., Behrens J., Meyer-Arndt L., Söder E., Brandt A.U., Bellmann-Strobl J., Ruprecht K., Gold S.M., Haynes J.-D. (2017). Brain activity, regional gray matter loss, and decision-making in multiple sclerosis. Mult. Scler..

[100] Koini M., Filippi M., Rocca M.A., Yousry T., Ciccarelli O., Tedeschi G., Gallo A., Ropele S., Valsasina P., Riccitelli G. (2016). Correlates of Executive Functions in Multiple Sclerosis Based on Structural and Functional MR Imaging: Insights from a Multicenter Study. Radiology.

[101] Matias-Guiu J.A., Cortés-Martínez A., Montero P., Pytel V., Moreno-Ramos T., Jorquera M., Yus M., Arrazola J., Matías-Guiu J. (2018). Structural MRI correlates of PASAT performance in multiple sclerosis. BMC Neurol..

[102] Bellmann-Strobl J., Wuerfel J., Aktas O., Dorr J., Wernecke K.D., Zipp F., Paul F. (2009). Poor PASAT performance correlates with MRI contrast enhancement in multiple sclerosis. Neurology.

[103] Pardini M., Uccelli A., Grafman J.H., Yaldizli O., Mancardi G.L., Roccatagliata L. (2014). Isolated cognitive relapses in multiple sclerosis. J. Neurol. Neurosurg. Psychiatry.

[104] Jehna M., Langkammer C., Wallner-Blazek M., Neuper C., Loitfelder M., Ropele S., Fuchs S., Khalil M., Pluta-Fuerst A., Fazekas F. (2011). Cognitively preserved MS patients demonstrate functional differences in processing neutral and emotional faces. Brain Imaging Behav..

[105] Mike A., Strammer E., Aradi M., Orsi G., Perlaki G., Hajnal A., Sandor J., Banati M., Illes E., Zaitsev A. (2013). Disconnection mechanism and regional cortical atrophy contribute to impaired processing of facial expressions and theory of mind in multiple sclerosis: A structural MRI study. PLoS ONE.

[106] Calabrese P., Penner I.K. (2007). Cognitive dysfunctions in multiple sclerosis “a multiple disconnection syndrome”. J. Neurol..

[107] Henry A., Tourbah A., Chaunu M., Rumbach L., Montreuil M., Bakchine S. (2011). Social cognition impairments in relapsing-remitting multiple sclerosis. J. Int. Neuropsychol. Soc..

[108] Batista S., d’Almeida O.C., Afonso A., Freitas S., Macário C., Sousa L., Castelo-Branco M., Santana I., Cunha L. (2017). Impairment of social cognition in multiple sclerosis: Amygdala atrophy is the main predictor. Mult. Scler..

[109] Chalah M.A., Ayache S.S. (2017). Deficits in Social Cognition: An Unveiled Signature of Multiple Sclerosis. J. Int. Neuropsychol. Soc..

[110] Benedict R.H., Hulst H.E., Bergsland N., Schoonheim M.M., Dwyer M.G., Weinstock-Guttman B., Geurts J.J., Zivadinov R. (2013). Clinical significance of atrophy and white matter mean diffusivity within the thalamus of multiple sclerosis patients. Mult. Scler..

[111] Schoonheim M.M., Hulst H.E., Brandt R.B., Strik M., Wink A.M., Uitdehaag B.M.J., Barkhof F., Geurts J.J.G. (2015). Thalamus structure and function determine severity of cognitive impairment in multiple sclerosis. Neurology.

[112] Bisecco A., Rocca M.A., Pagani E., Mancini L., Enzinger C., Gallo A., Vrenken H., Stromillo M.L., Copetti M., Thomas D.L. (2015). Connectivity-based parcellation of the thalamus in multiple sclerosis and its implications for cognitive impairment: A multicenter study. Hum. Brain Mapp..

[113] Nelson F., Akhtar M.A., Zúñiga E., Perez C.A., Hasan K.M., Wilken J., Wolinsky J.S., Narayana P.A., Steinberg J.L. (2016). Novel fMRI working memory paradigm accurately detects cognitive impairment in multiple sclerosis. Mult. Scler..

[114] Eijlers A.J., Meijer K.A., Wassenaar T.M., Steenwijk M.D., Uitdehaag B.M., Barkhof F., Wink A.M., Geurts J.J., Schoonheim M.M. (2017). Increased default-mode network centrality in cognitively impaired multiple sclerosis patients. Neurology.

[115] Campbell J., Langdon D., Cercignani M., Rashid W. (2016). A randomised controlled trial of efficacy of cognitive rehabilitation in multiple sclerosis: A cognitive, behavioural, and MRI study. Neural Plast..

[116] Pinter D., Beckmann C., Koini M., Pirker E., Filippini N., Pichler A., Fuchs S., Fazekas F., Enzinger C. (2016). Reproducibility of resting state connectivity in patients with stable multiple sclerosis. PLoS ONE.

[117] Boutiere C., Rey C., Zaaraoui W., Le Troter A., Rico A., Crespy L., Achard S., Reuter F., Pariollaud F., Wirsich J. (2017). Improvement of spasticity following intermittent theta burst stimulation in multiple sclerosis is associated with modulation of resting-state functional connectivity of the primary motor cortices. Mult. Scler..

[118] Pareto D., Sastre-Garriga J., Alonso J., Galán I., Arévalo M.J., Renom M., Montalban X., Rovira À. (2018). Classic Block Design “Pseudo”-Resting-State fMRI Changes After a Neurorehabilitation Program in Patients with Multiple Sclerosis. J. Neuroimaging.

[119] Fisher R.L., Priore R.L., Jacobs L.D., Cookfair D.L., Rudick R.A., Herndon R.M., Richert J.R., Salazar A.M. (2000). Neuropsychological effects of interferón beta 1 a in relapsing multiple sclerosis. Ann. Neurol..

[120] Patti F., Amato M.P., Bastianello S., Caniatti L., Di Monte E., Ferrazza P., Goretti B., Gallo P., Brescia Morra V., Lo Fermo S. (2010). Effects of immunomodulatory treatment with subcutaneous interferon beta-1a on cognitive decline in mildly disabled patients with relapsing-remitting multiple sclerosis. Mult. Scler..

[121] Patti F., Morra V.B., Amato M.P., Trojano M., Bastianello S., Tola M.R., Cottone S., Plant A., Picconi O. (2013). Subcutaneous interferon β-1a may protect against cognitive impairment in patients with relapsing-remitting multiple sclerosis: 5-year follow-up of the COGIMUS study. PLoS ONE.

[122] Pliskin N.H., Hamer D.P., Goldstein D.S., Towle V.L., Reder A.T., Noronha A., Arnason B.G. (1996). Improved delayed visual reproduction test performance in multiple sclerosis patients receiving interferon beta-1b. Neurology.

[123] Edan G., Kappos L., Montalbán X., Polman C.H., Freedman M.S., Hartung H.-P., Miller D., Barkhof F., Herrmann J., Lanius V. (2014). Longterm impact of interferon beta-1b in patients with CIS: 8-year follow-up of BENEFIT. J. Neurol. Neurosurg. Psychiatry.

[124] Weinstein A., Schwid S.I., Schiffer R.B., McDermott M.P., Giang D.W., Goodman A.D. (1999). Neuropsychologic status in multiple sclerosis after treatment with glatiramer. Arch. Neurol..

[125] Weinstock-Guttman B., Galetta S.L., Giovannoni G., Havrdova E., Hutchinson M., Kappos L., O’Connor P.W., Phillips J.T., Polman C., Stuart W.H. (2012). Additional efficacy endpoints from pivotal natalizumab trials in relapsing-remitting MS. J. Neurol..

[126] Jacques F.H., Harel B.T., Schembri A.J., Paquette C., Bilodeau B., Kalinowski P., Roy R. (2016). Cognitive evolution in natalizumab-treated multiple sclerosis patients. MSJ.

[127] Riepl E., Pfeuffer S., Ruck T., Lohmannt H., Wiendl H., Meuth S.G., Johnen A. (2018). Alemtuzumab improves cognitive proccesing speed in active multiple sclerosis—A longitudinal observational study. Front. Neurol..

[128] Hauser S.L., Bar-Or A., Comi G., Giovannoni G., Hartung H.-P., Hemmer B., Lublin F., Montalban X., Rammohan K.W., Selmaj K. (2017). Ocrelizumab versus Interferon Beta-1a in Relapsing Multiple Sclerosis. N. Engl. J. Med..

[129] Krupp L.B., Christodoulou C., Melville P., Scherl W.F., MacAllister W.S., Elkins L.E. (2004). Donepezil improved memory in multiple sclerosis in a randomized clinical trial. Neurology.

[130] Krupp L.B., Christodoulou C., Melville P., Scherl W.F., Pai L.Y., Muenz L.R., He D., Benedict R.H., Goodman A., Rizvi S. (2011). Multicenter randomized clinical trial of donepezil for memory impairment in multiple sclerosis. Neurology.

[131] Lovera J.F., Frohman E., Brown T., Bandari D., Nguyen L., Yadav V., Stuve O., Karman J., Bogardus K., Heimburger G. (2010). Memantine for cognitive impairment in multiple sclerosis: A randomized placebo-controlled trial. Mult. Scler..

[132] Sumowski J.F., Chiaravalloti N., Erlanger D., Kaushik T., Benedict R.H.B., DeLuca J. (2011). L-amphetamine improves memory in MS patients with objective memory impairment. Mult. Scler..

[133] Broicher S.D., Filli L., Geisseler O., Germann N., Zörner B., Brugger P., Linnebank M. (2018). Positive effects of fampridine on cognition, fatigue and depression in patients with multiple sclerosis over 2 years. J. Neurol..

[134] Pavsic K., Pelicon K., Ledinek A.H., Sega S. (2015). Short-term impact of fampridine on motor and cognitive functions, mood and quality of life among multiple sclerosis patients. Clin. Neurol. Neurosurg..

[135] Ford-Johnson L., DeLuca J., Zhang J., Elovic E., Lengenfelder J., Chiaravalloti N.D. (2016). Cognitive effects of modafinil in patients with multiple sclerosis: A clinical trial. Rehabil. Psychol..

[136] Millera E., Morelc A., Redlickaa J., Millera I., Salukc J. (2018). Pharmacological and Non-pharmacological Therapies of Cognitive Impairment in Multiple Sclerosis. Curr. Neuropharmacol..

[137] Chiaravalloti N.D., Moore N.B., Nikelshpur O.M., DeLuca J. (2013). An RCT to treat learning impairment in multiple sclerosis: The MEMREHAB trial. Neurology.

[138] Amato M.P., Goretti B., Viterbo R.G., Portaccio E., Niccolai C., Hakiki B., Iaffaldano P., Trojano M. (2014). Computer-assisted rehabilitation of attention in patients with multiple sclerosis: Results of a randomized, double-blind trial. Mult. Scler. J..

[139] De Giglio L., De Luca F., Prosperini L., Borriello G., Bianchi V., Pantano P., Pozzilli C. (2015). A low-cost cognitive rehabilitation with a commercial video game improves sustained attention and executive functions in multiple sclerosis: A pilot study. Neurorehabil. Neural Repair..

[140] Goverover Y., Chiaravalloti N.D., O’Brien A.R., DeLuca J. (2018). Evidenced-Based Cognitive Rehabilitation for Persons with Multiple Sclerosis: An Updated Review of the Literature From 2007 to 2016. Arch. Phys. Med. Rehabil..

[141] Carter A., Daley A., Humphreys L., Snowdon N., Woodroofe N., Petty J., Roalfet Al., Tosh J., Sharrack B., Saxton J. (2014). Pragmatic intervention for increasing self-directed exercise behaviour and improving important health outcomes in people with multiple sclerosis: A randomised controlled trial. Mult. Scler..

[142] Briken S., Gold S.M., Patra S., Vettorazzi E., Harbs D., Tallner A., Ketels G., Schulz K.H., Heesen C. (2014). Effects of exercise on fitness and cognition in progressive MS: A randomized, controlled pilot trial. Mult. Scler..

[143] Sandroff B.M., Motl R.W., Scudder M.R., DeLuca J. (2016). Systematic, Evidence-Based Review of Exercise, Physical Activity, and Physical Fitness Effects on Cognition in Persons with Multiple Sclerosis. Neuropsychol. Rev..

[144] Cramer H., Lauche R., Azizi H., Dobos G. (2014). ; Langhorst J Yoga for Multiple Sclerosis: A Systematic Review and Meta-Analysis. PLoS ONE.

